# Trends in Adolescent Substance Use: Analysis of HBSC Data for Four Eastern European Countries, 1994–2018

**DOI:** 10.3390/ijerph192315457

**Published:** 2022-11-22

**Authors:** Tomas Vaičiūnas, Monika Žemaitaitytė, Shannon Lange, Mindaugas Štelemėkas, Leila Oja, Janina Petkevičienė, Anna Kowalewska, Iveta Pudule, Jaanika Piksööt, Kastytis Šmigelskas

**Affiliations:** 1 Health Research Institute, Faculty of Public Health, Medical Academy, Lithuanian University of Health Sciences, 44307 Kaunas, Lithuania; 2 Centre for Addiction and Mental Health, Institute for Mental Health Policy Research, Toronto, ON M5S 2S1, Canada; 3 Centre for Addiction and Mental Health, Campbell Family Mental Health Research Institute, Toronto, ON M5G 2C1, Canada; 4 Department of Psychiatry, University of Toronto, Toronto, ON M5S 1A4, Canada; 5 National Institute for Health Development, 11619 Tallinn, Estonia; 6 Department of Biomedical Aspects of Development and Sexology, Faculty of Education, Warsaw University, 00-927 Warsaw, Poland; 7 Centre for Disease Prevention and Control, 1005 Riga, Latvia

**Keywords:** adolescent, substance use, trends, eastern Europe, HBSC

## Abstract

The aim of the study was to analyze the trends of adolescent substance use in four eastern European countries over the time period from 1994 to 2018. The four countries in focus were selected based on their shared historical backgrounds and major economic and social transformations experienced. Methods: Two decades (1993/1994–2017/2018) of repeated cross-sectional data from the Estonian, Latvian, Lithuanian, and Polish Health Behaviour in School-aged Children survey were used. Data comprised 42,169 school children 15 years of age (9th grade). The following categories of substance use were included: regular alcohol consumption and drunkenness, tobacco smoking, electronic cigarette smoking, and cannabis use. Trends in substance use over time were tested using Jonckheere’s trend test. Results: Prevalence of substance use among adolescents over time revealed that the Baltic states and Poland have faced relatively different temporal trends. In the Baltic states, there was a general increase during the period of 1994–2002, which was followed by a period of peaking or stability between 2002–2010, and then decreasing trends of these risky behaviors from 2010 onwards. In Poland, the same period had less consistent patterns, with decreasing trends starting much earlier on. The prevalence of cannabis use, which had been measured since 2006, had its own unique pattern with many fluctuations within and between countries. Conclusions: The findings on the prevalence of substance use among adolescents from 1994 to 2018 revealed that the Baltic states and Poland have faced relatively different temporal trends. These countries might be facing new public health challenges in a near future, e.g., use of electronic cigarettes and cannabis use among adolescents.

## 1. Introduction

In Europe, tobacco, alcohol, and illicit drug use contribute significantly to the burden of disease, ranking second, fifth, and twenty-second in terms of disability-adjusted life-years lost [1]. Adolescence is a time of experimentation with new behaviors such as substance use, which can ultimately shape an individual’s life course. As such, the health and well-being of young people is of particular concern, and efforts to reduce all types of risky behaviors among adolescents, including the consumption of tobacco, alcohol and illicit drugs, are ongoing [1,2].

Cigarettes are one of the most widely used legal substances, and adolescence is a crucial age for the development of smoking-related attitudes and behaviors, either through quitting or starting use of these substances [3,4]. Smoking has direct adverse health effects: among others, it is directly associated with addiction, impaired respiratory function, slower central nervous system function, and a direct impact on the expression of symptoms of depression and psycho-emotional fatigue [5]. Nicotine in cigarettes can damage the developing brain, affecting learning, memory, attention, and metabolic processes, and thus can increase the risk of addiction to other drugs and substances [5,6,7,8]. The last decade has seen an active change not only in the composition of tobacco products themselves, but also in the way they are presented and consumed, with the increasing popularity of e-cigarettes, vapes, etc. [5,6,8] which currently are lobbied by the tobacco industry as harm reduction products or for cigarette smoking cessation. However, the long-term effects of such products are largely unknown; they can act as a catalyst for smoking initiation, and likely cloud the reality of observed declining trends in tobacco smoking [9,10,11,12]. Current empirical studies show that e-cigarette use is linked to adverse physical and mental health outcomes [13], primary ingredients found in e-cigarettes (propylene glycol and vegetable glycerin) are toxic to cells, and that the more ingredients in an e-liquid, the greater the toxicity [14], and that the wide use of acrolein, a herbicide in e-cigarettes, could be linked to acute lung injury and chronic obstructive pulmonary disease, asthma, and lung cancer [15].

Adolescent alcohol consumption is one of the major public health concerns in many European countries [16]. Alcohol use has a complex association with health. Researchers have recognized alcohol use as a one of the leading risk factors for disease burden, and studies link its consumption to 60 acute and chronic diseases [3,17,18]. Thus, alcohol consumption during adolescence contributes significant health and social burdens. It is associated with an increased risk of both intentional and unintentional injuries, being involved in physical and sexual aggression/assaults, lower academic achievement, experiencing school-related negative consequences, and impaired brain development [18,19,20,21,22]. Furthermore, early alcohol use is also associated with lifetime struggles with addiction [23].

During the last decade, cannabis has become one of the most commonly used psychoactive drugs, particularly among adolescents and young adults. Relative to the general population, past-year prevalence rates of cannabis use are greatest among teenagers [21]. Heavy use is associated with an increased risk of cannabis use disorders [24], psychotic disorders, acute cognitive impairment [25], traffic injuries [26], respiratory problems, and poor pregnancy outcomes [27].

Overall, tobacco and alcohol use among youth are showing signs of decline in Europe, but there are concerns over potential increasing cannabis use [21]. In this article, we used the Health Behaviour in School-aged Children (HBSC) survey from four Eastern European Union (EU) countries (Estonia, Latvia, Lithuania, and Poland) to assess the temporal trends in tobacco use, alcohol consumption, and cannabis use among adolescents 15 years of age across seven consecutive survey waves covering a period of 1993–2018. The HBSC is coordinated by the World Health Organization (WHO) and consists of a self-reported questionnaire administered every four years [28].

The four countries in focus were selected based on their shared historical backgrounds and major economic and social transformations experienced. Specifically, all four countries are characterized to be similar in terms of Soviet past, major social and economic transformations after the early 1990s, their shared path to becoming members of international organizations, such as the EU in 2004, and various other societal challenges. On the other hand, they had some variability in other aspects that are worth acknowledging, e.g., the Baltic states were part of the Union of Soviet Socialist Republics, while Poland was not, and the Baltic states were greatly affected by the economic crisis in 2008–2010, while Poland was not. These countries are also experiencing similar challenges in terms of major public health indicators such as relatively lower life expectancy than the EU average and high levels of avoidable morality which is also closely related to the lifestyles developed by members of society in their early years of life. The aim of the current study was to analyze the trends of adolescent substance use in four Eastern European countries from 1994 to 2018. We analysed data for 15-year-olds only, because the patterns of substance use for adolescents 15 years of age, although still developing, are already more stable than those of younger adolescent age groups.

## 2. Materials and Methods

### 2.1. Sample and Procedure

The present data were drawn from the HBSC surveys. The HBSC is a WHO collaborative cross-national study which has been conducted every four years since 1984 and uses a standardised self-report survey. The overall aim of the study is to provide up-to-date information on the health, well-being, social environment, and health behaviors of 11-, 13-, and 15-year-old school children from participating countries (i.e., from 50 countries and regions across Europe, North America, and the Middle East) [28].

The current analysis used data from Estonia, Latvia, Lithuania, and Poland collected during 1993/94, 1997/98, 2001/02, 2005/06, 2009/10, 2013/14, and 2017/18 (referred to as 1994, 1998, 2002, 2006, 2010, 2014, and 2018, respectively, herein). To ensure a nationally representative sample, a randomised cluster probability sampling approach was carried out in all countries’ school classes, with the aim of obtaining 1500 respondents for each age group. The recommended sample size was calculated based on the expected design effect and to account for the effect of clustering [28]. Only data from the 15-year-old age group will be analysed in the current analysis (Table 1). The response rates in 2018 ranged from 74% to 90% (in previous survey years response rates were collected inconsistently).

Ethical permissions for the surveys were obtained at the national level. Participation was voluntary and the children as well as their parents (either one or both) were informed about confidentiality and anonymity. Data collection follows a standard methodology outlined in the HBSC protocol [28].

### 2.2. Measures

For the current analysis, only the questions on alcohol, tobacco, and cannabis use that remained consistent from 1994 to 2018 were selected and analysed, given that the wording of the respective questions and their answer options did not change or only changed slightly. Questions on e-cigarettes were only added in 2018 because of the novelty of the product itself. The consistency of items for Estonia, Latvia, Lithuania, and Poland was also considered in this study (Table 2).

Five different types of substance use were included in the analysis: regular alcohol consumption and drunkenness, smoking (cigarettes and electronic cigarettes), and cannabis use.

Tobacco smoking was measured by asking participants how often they smoke tobacco. Response options were on a 4-point scale from ‘every day’, ‘at least once a week, but not every day’, ‘less than once a week’ to ‘I do not smoke’. The findings from the data analysis are the proportion of 15-year-olds who reported smoking at least once a week (regular smoking).

Electronic cigarette smoking was assessed by asking participants how many days (if any) in the last 30 days they had used electronic cigarettes. Response options were ranging from ‘never’, ‘1–2 days’, ‘3–5 days’, ‘6–9 days’, ’10–19 days’, ’20–29 days’ to ‘30 days’. The findings from the data analysis are the proportion of 15-year-olds who had used an electronic cigarette at least once in the last 30 days.

Alcohol consumption was assessed by asking participants how often they drank anything alcoholic such as beer, wine, spirits, or alcopops, including even those times when they drank only a small amount. Participants were asked to report the frequency of each type of alcohol they consumed with the following response options: ‘every day’, ‘every week’, ‘every month’, ‘rarely’, and ‘never’. The findings from the data analysis are the proportion of 15-year-olds who had used alcohol at least once a week (regular use of any alcoholic drink). The 2018 data from Poland was not available. A derived indicator on regular drinking was obtained by using the responses to the questions on the consumption of different alcoholic beverages, i.e., those adolescents who drank any type of alcohol at least once a week.

Drunkenness was assessed by asking participants how many times in their lifetime they drank so much alcohol that they were really drunk. Response options were on a 5-point scale from ‘never’, ‘yes, once’, ‘yes, 2–3 times’, ‘yes, 4–10 times’ to ‘yes, more than 10 times’. In this study, the drunkenness among pupils in four countries was defined by comparing responses indicating drunkenness 2 or more times.

Lastly, cannabis use was assessed by asking participants on how many days (if any) in the last 30 days they had used cannabis. The response options ranged from ‘never’, ‘1–2 days’, ‘3–5 days’, ‘6–9 days’, ’10–19 days’, ’20–29 days’ to ‘30 days’. The data analysis focused on adolescents who had used cannabis at least once in the previous 30 days.

### 2.3. Statistical Analysis

Data were processed using MS Excel 2016 and analysed using IBM SPSS Statistics for Windows, Version 26.0 [29]. All analyses were stratified by sex (i.e., boys and girls). Survey weights were used in all analyses. Equal weighting was given to all countries in the computed averages across the Baltic states. Trends in smoking, alcohol consumption and cannabis use over time have been tested using Jonckheere’s trend test. In Jonckheere’s trend test, the alternative hypothesis is tested against the null hypothesis that there is no systematic trend in the response groups [30]. Smoking prevalence of both cigarettes and e-cigarettes between the Baltic states and Poland have been tested using Pearson’s chi-square test. A statistical significance level of α = 0.05 was used.

## 3. Results

### 3.1. Cigarette Smoking

**Figure 1:**
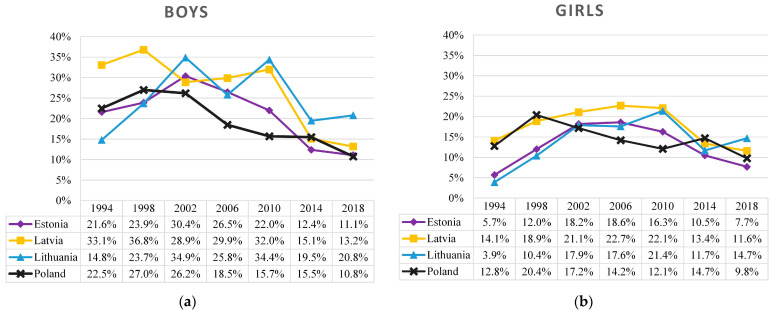
Prevalence of regular cigarette smoking (at least once a week) among 15-year-old boys (**a**) and girls (**b**) in the Baltic states and Poland, 1994–2018.

Figure 1 describes the prevalence of regular (weekly or more frequent) cigarette smoking among 15-year-old adolescents. Here, all four countries showed quite different trends from 1994 to 2018. Until 2002, growth in regular smoking among boys was observed in Lithuania (15–35%) and less expressed in Estonia (22–30%), while in Poland the situation remained stable (26–27%). In Latvia, meanwhile, fluctuations were observed. Since 2002, prevalence of regular smoking decreased sharply in Poland (26% to 11%) and Estonia (30% to 11%); however, in Lithuania and Latvia the elevated rate lasted until 2010 and only then adolescent smoking has decreased (from 34 to 21% and from 34% to 13%, respectively).

The trend and breakpoints were more similar among girls. In the Baltic states, there was a period of increasing tobacco smoking prevalence from 1994 (4–14%) to 2002 (18–21%), then the situation was relatively stable until 2010 (16–22%), and later it decreased until 2018 (8–15%). In comparison, the studied period in Poland followed a different pattern: it increased until 1998 (13–20%) and then gradually decreased with minor fluctuations until 2014 (from 20% to 10%).

Between 1994 and 2018 in Poland, the overall percentage of boys who regularly smoked cigarettes decreased by more than two times (T_JT_ = 2.00, z = −2.553, *p* = 0.011), while among girls it slightly varied (T_JT_ = 6.00, z = −1.352, *p* = 0.176). The trends of the tobacco smoking prevalence among girls were quite similar in all Baltic states: an increase in smoking until 2002 (T_JT_ = 23.00, z = 2.111, *p* = 0.035), a plateau phase from 2002 to 2010, and a slight decrease since 2010 (T_JT_ = 4.00, z = −2.111, *p* = 0.035). In recent surveys (2014–2018), the prevalence of smoking among boys and girls has been similar (about 15%) in all four countries, with larger gender differences observed in Lithuania (21% in boys and 15% in girls).

Given that e-cigarette use is a relatively recent phenomena, only the survey in 2018 obtained data on e-cigarette use (Table 3). Here, the highest prevalence of e-cigarette use was observed in Lithuania, with less than one eighth of boys and girls (12%) reporting e-cigarettes use at least once in the last month and almost a quarter of boys (23%) and one seventh of girls (14%) reporting electronic use and conventional cigarettes smoking at the same time. In the other Baltic states, the frequency of only e-cigarette use among boys is similar (Estonia—12%, Latvia—11%; e-cigarette use is less common among girls (Estonia—7%, Latvia—5%)). In Poland, e-cigarettes were used by 10% of 15-year-olds. Overall smoking prevalence in Poland was the lowest across all analyzed countries.

With respect to the overall smoking frequency of both cigarettes and e-cigarettes, the highest prevalence was observed in Lithuania, with almost half of the boys (43%) and more than a third of the girls (39%) reporting smoking at least once in the last month. In the other Baltic states, about one in three boys (Estonia—31%; Latvia—32%) and one in four girls (Estonia—23%; Latvia—29%) have smoked in the last month. In Poland, about a quarter of 15-year-olds smoked (26% of boys and 27% of girls).

### 3.2. Regular Alcohol Consumption

**Figure 2:**
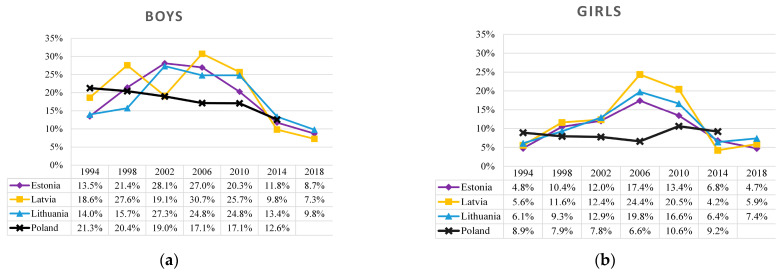
Prevalence of regular alcohol consumption (consumption of any type of alcohol at least once a week) among 15-year-old boys (**a**) and girls (**b**) in the Baltic states and Poland, 1994–2018.

Figure 2 describes the prevalence of regular (weekly or more frequent) alcohol consumption in Estonia, Latvia, Lithuania, and Poland among 15-year-old boys and girls from 1994 to 2018. During this period, trends in regular alcohol consumption were largely different in the Baltic states and Poland. Among boys in the Baltic states, the prevalence of regular alcohol consumption varied from 14 to 19% in 1994, peaked at 25–31% between 2006 and 2010, and then decreased to 7–10% in 2018, while among girls, the prevalence was quite similar across all the years (i.e., from 5–6% in 1994 to 5–7% in 2018) but had a clearly defined peak of 17–24% in 2006. Meanwhile, in Poland during the study period, the trend was consistently declining among boys (from 21% to 13%) but not among girls.

In the Baltic states, there were relatively similar trends in regular drinking of boys and girls from 1994 to 2018, though the inflection point was slightly different. Among boys, the breakpoint was between 2002 and 2010: the first inflection point was observed in Estonia in 2002, then in Latvia in 2006, while in Lithuania the peak of regular alcohol consumption had plateaued from 2002 to 2010, meanwhile among girls in 2006 there was a clear breakpoint in the trend of regular alcohol consumption. In the Baltic states, regular alcohol consumption among boys had doubled during the study period (T_JT_ = 23.00, z = 2.111, *p* = 0.035; though in Latvia, the growth trend has been unstable), and among girls had tripled by 2006 (T_JT_ = 54.00, z = 3.838, *p* < 0.001). Following that, the trends shifted in the opposite direction: from 2006 to 2018, regular alcohol consumption among adolescents declined sharply, with about a threefold decrease among both boys (T_JT_ = 2.00, z = −3.568, *p* < 0.001) and girls (T_JT_ = 7.00, z = −2.843, *p* = 0.004).

In contrast, the analysed study period in Poland had a remarkably different trend pattern. Since 1994, there had been a relatively stable decline in regular drinking among boys (T_JT_ = 0.50, z = −2.68, *p* = 0.007), while among girls the decline was observed starting in 2006 (T_JT_ = 0.00, z = −2.038, *p* = 0.042). Thereafter, there had been an increase in alcohol consumption among girls that almost returned to the baseline level in 1994.

### 3.3. Drunkenness

**Figure 3:**
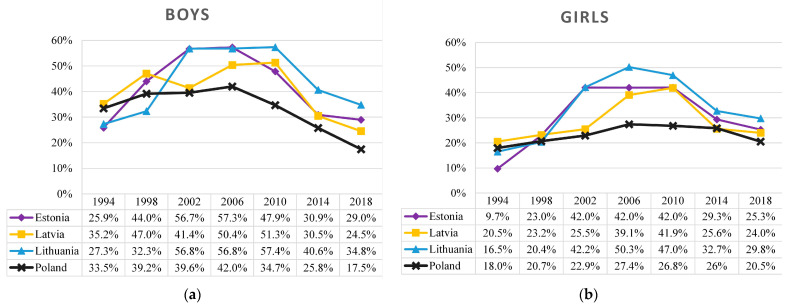
Prevalence of drunkenness (defined as having been drunk two or more times in their lifetime) among 15-year-old boys (**a**) and girls (**b**) in the Baltic states and Poland, 1994–2018.

The trends of drunkenness in the Baltic states and Poland were rather similar (Figure 3). In general, among boys there was a period of growth from 1994 (26–34%) to 2002 (40–57%), then the prevalence of drunkenness was relatively stable until 2010 (35–57%), and later it steeply decreased until 2018 (18–35%). The described trends of drunkenness were similar to those among girls (in comparison with boys), except for a remarkably lower baseline prevalence in 1994 (10–18%) and slightly lower peak at breakpoint (27–50%). Overall, the prevalence of drunkenness was lower in Poland compared to the Baltic states. 

When describing the trends, in Poland an inflection point was observed in 2006, while in the Baltic states the peak in the prevalence of drunkenness had plateaued between 2002 and 2010. From 1994 to 2006, the prevalence of drunkenness among 15-year-olds in Poland grew slower (among boys and girls T_JT_ = 6.00, z = 2.038, *p* = 0.042) than in the Baltic states from 1994 to 2002 (boys T_JT_ = 24.00, z = 2.333, *p* = 0.020; girls T_JT_ = 26.00, z = 2.778, *p* = 0.005). In Poland, the percentage of boys who have been drunk twice or more in their lifetime decreased from 2006 by almost two times (T_JT_ = 0.00, z = −2.038, *p* = 0.042), while the prevalence of drunkenness among girls had declined, but not at statistically significant levels (T_JT_ = 6.00, z = −1.81, *p* = 0.071). Meanwhile, in the Baltic states, adolescent drunkenness has decreased significantly, with boys and girls experiencing a reduction of more than two-fold (boys T_JT_ = 6.50, z = −2.920, *p* = 0.004; girls T_JT_ = 6.00, z = −2.985, *p* = 0.003).

### 3.4. Cannabis Use

**Figure 4:**
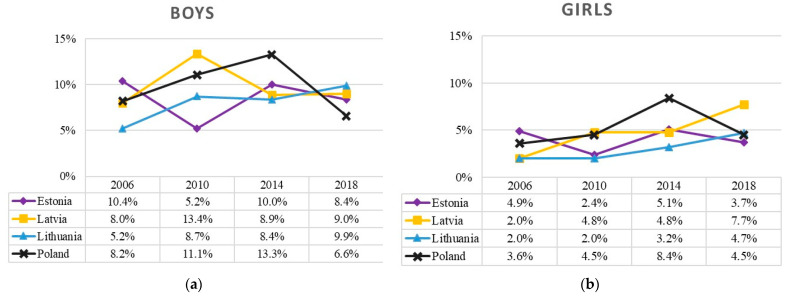
Prevalence of cannabis use (at least once in the last 30 days) among 15-year-old boys (**a**) and girls (**b**) in the Baltic states and Poland, 2006–2018.

Over the past decade, the trends of cannabis use during the 30-day period were relatively different in the Baltic states and Poland (Figure 4). The prevalence of cannabis use among boys fluctuated more (from 5% to 13%) than that of among girls (from 2% to 8%). The prevalence of cannabis use increased in the Baltic states (T_JT_ = 38.50, z = 1.651, *p* = 0.099), but remained stable in Poland with a sharper decrease after 2014 (4–8%, T_JT_ = 4.50, z = 1.083, *p* = 0.279), albeit not at statistically significant levels.

## 4. Discussion

Overall, the current findings on the prevalence of substance use among adolescents over time revealed that the Baltic states and Poland have faced relatively different temporal trends. In the Baltic states there was a general increase during the period of 1994–2002, which was followed by a period of peaking or stability between 2002–2010, and then decreasing trends of these risk behaviors from 2010 onwards. The same period in Poland displayed less consistent patterns, with decreasing trends starting much earlier on. The prevalence of cannabis use, which had been measured since 2006, had its own unique pattern with many fluctuations within and between countries.

When looking at the differing national trends of substance use among adolescents in the Baltic states and Poland over time, changes in national policies and legislations or regulations first come to mind. For instance, there may be important similarities as well as a natural split between Baltic states and Poland with respect to the implementation of alcohol control policies. Further, an important time point for all of the countries was May 2004 when all four countries joined the EU, resulting in a rapid economic growth which may have been associated with increased alcohol affordability and some reduction after 2007 [31,32]. In 2008–2010, the global economic crisis hit the Baltic states significantly resulting in a major drop in per capita GDP (a drop by 15%, 18% and 14% from 2008 to 2009 in Lithuania, Latvia and Estonia, respectively), while Poland had avoided a major economic setback with no decline in GDP [33]. In addition, at the beginning of the economic crisis period, all the governments from the four countries were motivated to increase taxation in 2008–2009 (an increase in excise taxation by 10–20% since 2008 in Estonia, by 33–62% in Latvia since 2009, by 10–20% in 2008 and again in 2009 in Lithuania, and by 9–16% in Poland) [34,35]. Some additional alcohol control policies were introduced in Estonia (off-premise sale ban from 10 p.m. to 10 a.m. since 2008; some additional marketing restrictions). In terms of physical availability of alcohol in Estonia, more attention has been paid to cross-border alcohol trade [31]. In Lithuania, the period of economic crisis has overlapped with a significant shift in a societal and political view towards alcohol due to significant harm of alcohol in the society, and a shift in alcohol control legislation was observed since 2008 declaring ‘the year of sobriety’ and introducing several strong alcohol control policies (ban of alcohol advertising on radio and TV during the day time 6 a.m. to 11 p.m. since 2008; restricted off-premise sales from 10 p.m. to 8 a.m. since 2009) [35]. At the individual level, an additional initiative for early identification and counselling of alcohol-related health problems was launched in primary health care in the beginning of 2010 [36]. However, no other strong alcohol policies were introduced in Latvia. In 1990, many legal measures concerning the health of adults, children and adolescents were introduced in Poland. Since then, there has been a sharp improvement in alcohol drinking and smoking rates, but this did not stop for alcohol in 2002. In the beginning of the 21st century, a weakening of the Polish government’s alcohol control policy has been observed. In 2002, the excise tax on spirits was reduced by 30%, in 2001 the beer regulations were suspended. Beer advertising was re-introduced on television. In addition, in 2010 the alcohol industry launched an ongoing marketing campaign leading to a dramatic increase in sales of small bottles of vodka [37].

With respect to the trends of regular alcohol drinking and drunkenness, they may be associated with a lack of restrictive alcohol control policies since the Baltic states were preparing to join and joined the EU in 2004 up till 2007, while since 2008 a period of lower alcohol availability (in terms of restrictions on marketing, physical availability, and increases in price) had started. The HBSC surveys have indicated that between 2006 and 2010 the alcohol drinking among boys and girls tended to stop growing and during the last two survey rounds (2014 and 2018) a shift towards declining trends was observed.

Any associations of adolescent smoking with the evidence-based tobacco control policies may be more complex due to emerging popularity of nicotine delivery systems such as electronic cigarettes [38]. However, the important time point for all the countries was the ratified Framework Convention for Tobacco Control (FCTC) which was approved by the World Health Organization (WHO) in 2003–2004 [39], which has introduced many marketing and availability restrictions. It may have benefited by declining smoking prevalence trends among boys and partly among girls since 2004; however, when observing a more rapid decline in tobacco smoking prevalence since the 2014 survey, it demands a more careful look due to an increasing popularity of smoking alternatives (e.g., e-cigarettes). For example, in 2014, in Lithuania, the Law on Tobacco Control has been amended with additional restrictions (including possession restrictions) on sales of electronic cigarettes and refillable containers to underage users [40]. In Estonia, the revision of the Tobacco Act [41] in 2015 defined e-cigarettes as tobacco products and established several restrictions to the use of e-cigarettes and refill liquids. Since 2018, similar to smoking, the use of e-cigarettes in public spaces is also prohibited. In Latvia, the Law on Handling Tobacco Products was substantially changed in 2016, defining e-cigarettes and establishing similar restrictions as for tobacco cigarettes [42]. The HBSC survey currently provides only one cross-sectional time point of data on e-cigarettes to compare the four countries, and thus, the change in smoking prevalence needs to be closely monitored in the future HBSC studies.

Despite cannabis being illegal to use, sell and buy in all four countries, it is clearly still being used, albeit by a relatively small proportion of adolescents. Regardless, its use indicates a lack of or ineffective cannabis control policies and enforcement on the national level, and its growing popularity in some countries (e.g., Lithuania) suggest that attention is needed to address this public health concern. 

### Study Limitations

To the best of our knowledge, this is the first study to investigate trends in alcohol consumption, tobacco smoking, e-cigarette smoking and cannabis use among adolescents across four Eastern European countries. However, there are a few limitations that should be noted. First, the current study utilized self-reported data, which has some inherent limitations that are important to note, i.e., social desirability bias and recall bias could have had an impact on the responses provided and thus may have impacted the estimates presented here. Moreover, the study involves a 24-year period during which the social generation of children has changed (including social desirability trends), which was not possible to assess in our study, but may have had an impact on the likelihood of reporting certain behaviors. Lastly, the HBSC survey is implemented once every four years, meaning that it is not a continuous annual series which may result in a lower precision when representing the impact of events such as an economic crisis or specific public health policies introduced in a specific year.

## 5. Conclusions

Findings on the prevalence of substance use among adolescents from 1994 to 2018 revealed that the Baltic states and Poland have faced relatively different temporal trends. Prevalence of regular (weekly or more frequent) smoking was different between countries, with a gender difference noted, i.e., higher among boys than girls. From 1994 to 2002, growth in regular smoking among boys was observed in Lithuania, less expressed in Estonia, similar in Latvia, while in Poland the situation remained stable. Since 2002, the regular smoking decreased sharply in Poland and Estonia; however, in Lithuania and Latvia the peak lasted until 2010 and only then adolescent smoking has decreased. Among girls, in Baltic states, there was a period of increasing tobacco smoking prevalence from 1994 to 2002, then the situation was relatively stable until 2010, and later it decreased until 2018. Between 2014 and 2018, the prevalence of smoking among boys and girls has been similar in all four countries, with larger gender differences observed in Lithuania. The prevalence of regular alcohol consumption among boys in Baltic states was slightly increasing from 1996 and peaked in 2006. In the period from 2006 to 2018, prevalence of alcohol consumption was decreasing rapidly, while among girls during the same period the prevalence was quite similar throughout years, but had a clearly defined peak in 2006. Over the past decade, Baltics and Poland have been facing a new public health challenge—electronic cigarette smoking. Overall, the findings suggest that more needs to be done in the Baltic states and Poland to address the use of substances among adolescents, most notably alcohol and cigarettes.

## Figures and Tables

**Table 1:** Sample size of adolescents, 15 years of age, by country and survey year.

		Survey Year						
	Country	1993/94	1997/98	2001/02	2005/06	2009/10	2013/14	2017/18
Sample size (*n*)	Estonia	1179	587	1267	1587	1398	1269	1542
	Latvia	1263	1265	1117	1330	1375	1726	1342
	Lithuania	1759	1435	1905	1861	1792	1698	1182
	Poland	1540	1636	2152	2287	1410	1484	1781

**Table 2:** Substance use measurements across time.

Substance	Period of Use	Survey Year
Cigarette smoking	at present	1994–2018
Electronic cigarette smoking	last 30 days	2018
Alcohol consumption	at present	1994–2018
Drunkenness	lifetime	1994–2018
Cannabis use	last 30 days	2006–2018

**Table 3:** Prevalence of smoking (at least once a month) among 15-year-olds in the Baltic states and Poland, 2018.

Country	Gender	Total Smoking Prevalence	Tobacco Cigarette Only	Only Electronic Cigarette Using	Electronic and Tobacco Cigarette	X^2^	*p*
Estonia	Boys	31.3	8.9	11.9	10.5	91.80	<0.001
	Girls	23.3	7.6	6.9	8.8		
	Boys + girls	27.2	8.2	9.4	9.6		
Latvia	Boys	31.9	8.7	10.9	12.3		
	Girls	28.6	15.0	5.2	8.4		
	Boys + girls	30.3	11.9	8.0	10.3		
Lithuania	Boys	42.7	7.6	12.2	22.9		
	Girls	39.1	13.4	12.0	13.7		
	Boys + girls	40.8	10.6	12.1	18.1		
Poland	Boys	25.8	6.1	9.3	10.4		
	Girls	27.1	8.7	7.8	10.6		
	Boys + girls	26.5	7.5	8.5	10.5		

## Data Availability

The data were provided by the HBSC Data Management Center at the University of Bergen, Norway, to which requests for data should be submitted.
